# Supplementary material for: Combination of Conventional Drugs with Biocompounds Derived from Cinnamic Acid: A Promising Option for Breast Cancer Therapy
Source: Biomedicines. 2023 Jan 19;11(2):275. doi: 10.3390/biomedicines11020275 (PMC9952910; doi:10.3390/biomedicines11020275)
Supplement: Supplementary file 1 [file biomedicines-11-00275-s001.zip › biomedicines-2149610-supplementary.pdf]

Supplementary File(s)

Evaluation risk of bias

The risk of bias was analyzed for the 9 selected publications available in Figure S1. The exclusively in vitro trials or with a part in vitro [37–45] presented between 80–100% of the domains for low risk of bias (scientific background and rationale, objectives and/or hypotheses, the intervention of each group, outcomes, sample size, statistical methods, outcomes and estimation and funding). On the other hand, these publications pointed to a high risk of bias domains (randomization – sequence generation, allocation concealment mechanism, implementation, blinding, limitations and protocol) that have not been reported in 92–100% of in vitro or with a part in vitro studies. No publication selected in the systematic review presented all domains for identifying the risk of bias.

Studies	Checklist item														
	1	2a	2b	3	4	5	6	7	8	9	10	11	12	13	14
Suberu et al 2014	-	+	+	+	+	+	-	-	-	-	-	-	-	+	-
Motawi et al 2016	-	+	+	+	+	+	-	-	-	-	+	+	-	+	-
Motawi et al (1) 2016	-	+	+	+	+	+	-	-	-	-	+	-	-	+	-
Fouad et al 2021	+	+	+	+	+	+	-	-	-	-	+	+	-	+	-
Zheng et al 2015	-	+	+	+	+	+	-	-	-	-	+	+	-	+	-
Choi and Park 2015	-	?	?	+	+	+	-	-	-	-	+	+	-	-	-
Torki et al 2017	-	+	+	+	+	+	-	-	-	-	+	+	-	+	-
Islam et al 2017	-	+	+	+	+	+	?	-	-	-	+	+	-	+	-
Carranza-Torres et al 2015	+	+	?	+	+	+	-	-	-	-	+	+	-	+	-

1) Structured abstract; 2a) Scientific background and rationale; 2b) Objectives and/or hypotheses; 3) Intervention of each group; 4) Outcomes; 5) Sample size; 6) Randomization: sequence generation; 7) Allocation concealment mechanism; 8) Implementation; 9) Blinding; 10) Statistical methods; 11) Outcomes and estimation; 12) Limitations; 13) Funding; 14) Protocol. (+) Low risk of bias; (-) High risk of bias; (?) Unclear risk of bias.

Figure S1. Risk of bias of the in vitro studies according to the modified CONSORT checklist.

Table S1. Databases and search strategy used, and numbers of retrieved studies.

Database	Search strategy	Hits
PUBMED searched at December 31, 2022	#1Breast Neoplasms<br><br>#2Cinnamates OR phenolic acid [Supplementary Concept] OR Phenylpropionates<br><br>#3Drug Synergism OR Drug Therapy, Combination OR Drug Combinations	17
WEB OF SCIENCE searched at December 31, 2022	#1ALL=(Breast cancer)<br><br>#2ALL=((Cinnamates) OR (Phenolic acid) OR (Phenylpropionates))<br><br>#3ALL=((Drug Synergism) OR (Drug Therapy, Combination) OR (Drug Combinations))	17
EMBASE searched at December 31, 2022	#1'breast tumor'<br><br>#2'cinnamic acid derivative' OR 'phenolic acid' OR 'phenylpropionic acid derivative'<br><br>#3'drug potentiation' OR 'combination drug therapy' OR 'drug combination'	4
SCOPUS searched at December 31, 2022	(ALL ("Breast Neoplasms") AND ALL ("Cinnamates" OR "phenolic acid" OR "Phenylpropionates") AND ALL ("Drug Synergism" OR "Drug Therapy, Combination" OR "Drug Combinations"))	11
LILACS searched at December 31, 2022	#1Breast Neoplasms<br><br>#2Cinnamates OR phenolic acid OR Phenylpropionates<br><br>#3Drug Synergism OR Drug Therapy, Combination OR Drug Combinations	0
COCHRANE searched at December 31, 2022	#1"Breast Neoplasms"<br><br>#2"Cinnamates" OR "phenolic acid" OR "Phenylpropionates"<br><br>#3"Drug Synergism" OR "Drug Therapy, Combination" OR "Drug Combinations"	0

Table S2. Studies included in the systematic review.

Carranza-Torres, I.E.; Guzmán-Delgado, N.E.; Coronado-Martínez, C.; Bañuelos-García, J.I.; Viveros-Valdez, E.; Morán-Martínez, J.; Carranza-Rosales, P. Organotypic Culture of Breast Tumor Explants as a Multicellular System for the Screening of Natural Compounds with Antineoplastic Potential. *Biomed Res. Int.* **2015**, *2015*. https://doi.org/10.1155/2015/618021.
Choi, Y.E.; Park, E. Ferulic Acid in Combination with PARP Inhibitor Sensitizes Breast Cancer Cells as Chemotherapeutic Strategy. *Biochem. Biophys. Res. Commun.* **2015**, *458*, 520–524. https://doi.org/10.1016/J.BBRC.2015.01.147.
Fouad, M.A.; Sayed-Ahmed, M.M.; Huwait, E.A.; Hafez, H.F.; Osman, A.M.M. Epigenetic Immunomodulatory Effect of Eugenol and Astaxanthin on Doxorubicin Cytotoxicity in Hormonal Positive Breast Cancer Cells. *BMC Pharmacol. Toxicol.* **2021**, *22*, 1–15. https://doi.org/10.1186/s40360-021-00473-2.
Islam, S.S.; Al-Sharif, I.; Sultan, A.; Al-Mazrou, A.; Remmal, A.; Aboussekhra, A. Eugenol Potentiates Cisplatin Anti-Cancer Activity through Inhibition of ALDH-Positive Breast Cancer Stem Cells and the NF-KB Signaling Pathway. *Mol. Carcinog.* **2018**, *57*, 333–346. https://doi.org/10.1002/MC.22758.
Motawi, T.K.; Abdelazim, S.A.; Darwish, H.A.; Elbaz, E.M.; Shouman, S.A. Modulation of Tamoxifen Cytotoxicity by Caffeic Acid Phenethyl Ester in MCF-7 Breast Cancer Cells. *Oxid. Med. Cell. Longev.* **2016**, *2016*. https://doi.org/10.1155/2016/3017108.
Motawi, T.K.; Abdelazim, S.A.; Darwish, H.A.; Elbaz, E.M.; Shouman, S.A. Could Caffeic Acid Phenethyl Ester Expand the Antitumor Effect of Tamoxifen in Breast Carcinoma? *Nutr. Cancer* **2016**, *68*, 435–445. https://doi.org/10.1080/01635581.2016.1153669.
Suberu, J.O.; Romero-Canelón, I.; Sullivan, N.; Lapkin, A.A.; Barker, G.C. Comparative Cytotoxicity of Artemisinin and Cisplatin and Their Interactions with Chlorogenic Acids in MCF7 Breast Cancer Cells. *ChemMedChem* **2014**, *9*, 2791–2797. https://doi.org/10.1002/CMDC.201402285.
Torki, S.; Soltani, A.; Shirzad, H.; Esmaeil, N.; Ghatrehsamani, M. Synergistic Antitumor Effect of NVP-BEZ235 and CAPE on MDA-MB-231 Breast Cancer Cells. *Biomed. Pharmacother.* **2017**, *92*, 39–45. https://doi.org/10.1016/j.biopha.2017.05.051.
Zheng, X.; Chen, S.; Yang, Q.; Cai, J.; Zhang, W.; You, H.; Xing, J.; Dong, Y. Salvianolic Acid A Reverses the Paclitaxel Resistance and Inhibits the Migration and Invasion Abilities of Human Breast Cancer Cells by Inactivating Transgelin 2. *Cancer Biol. Ther.* **2015**, *16*, 1407. https://doi.org/10.1080/15384047.2015.1070990.
